# P-694. Outcomes of respiratory viral infections in hematopoietic cell transplant recipients and patients with leukemia: a prospective study

**DOI:** 10.1093/ofid/ofae631.890

**Published:** 2025-01-29

**Authors:** Georgios Angelidakis, Tali Shafat, Layale Yaghi, Fareed Khawaja, Flavio Pignataro Oshiro, Joshua M Kirsch, Ella Ariza Heredia, Marjorie Batista, Kenneth J Gollob, Breck A Duerkop, Roy F Chemaly, Amy Spallone

**Affiliations:** The University of Texas Md Anderson Cancer Center, Houston, Texas; The University of Texas MD Anderson Cancer Center, Houston, Texas; UT MD Anderson Cancer CEnter, Houston, Texas; The University of Texas MD Anderson Cancer Center, Houston, Texas; A.C.Camargo Cancer Center, Sao Paulo, Sao Paulo, Brazil; University of Colorado Anschutz Medical Center, Aurora, Colorado; The University of Texas MD Anderson Cancer Center, Houston, Texas; Department of Infectious Diseases, AC Camargo Cancer Center, São Paulo, SP, Brazil., São Paulo, Sao Paulo, Brazil; Albert Einstein Israelite Hospital, Sao Paulo, Sao Paulo, Brazil; University of Colorado Anschutz Medical Campus, Aurora, Colorado; University of Texas MD Anderson Cancer Center, Houston, TX; University of Texas MD Anderson Cancer Center, Houston, TX

## Abstract

**Background:**

Respiratory viral infections (RVIs) pose a significant clinical challenge in immunocompromised hosts, including patients with leukemia and hematopoietic cell transplant (HCT) recipients. Despite advances in infection prevention, diagnostics, and clinical management, RVIs continue to be a major cause of morbidity and mortality in this patient population, due to progression to lower respiratory tract infection (LRTI). We aimed to identify risk factors associated with progression to LRTI in patients with leukemia and HCT recipients.

**Table 1:**
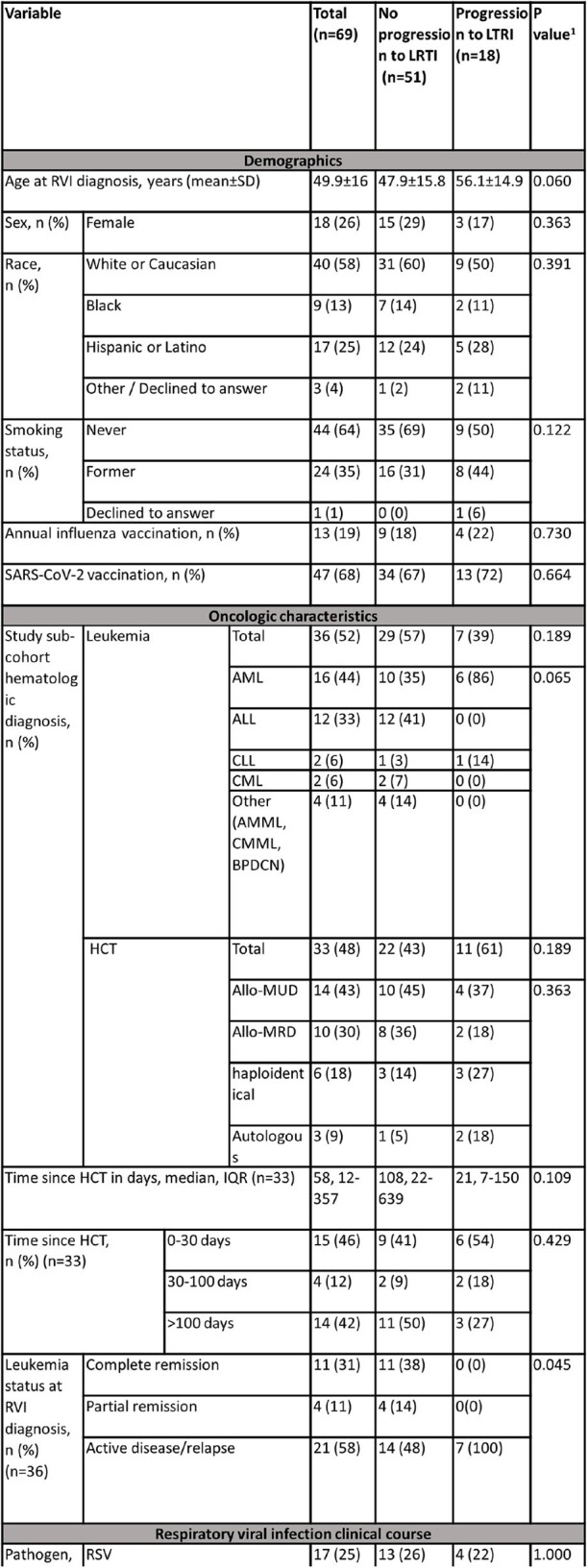
Baseline characteristics and clinical outcomes of leukemia and Hematopoietic Cell Transplantation patients at enrollment with upper respiratory viral infections versus controls. 1Continuous variables were compared using one-way ANOVA or Kruskal-Wallis rank sum test, and categorical data were analyzed using χ2 or Fisher's exact tests, as appropriate. P-value <0.05 was considered as statistically significant. Abbreviations: SARS-Cov, severe-acute-respiratory-syndrome-related coronavirus; AMML, acute myelomonocytic leukemia; CMML, chronic myelomonocytic leukaemia; BPDCN, blastic plasmacytoid dendritic cell neoplasm; AML, acute myeloid leukemia; MRD, matched related donor; MUD, matched unrelated donor; RSV, respiratory syncytial virus; IVIG, intravenous immunoglobulin; HFNC, high frequency nasal canula; GVHD, graft versus host disease.

**Methods:**

From July 2021 through January 2024, we conducted a prospective observational study of patients with leukemia and HCT recipients. We included patients with RVI symptoms, a positive respiratory viral panel for respiratory syncytial virus (RSV), influenza, or parainfluenza (PIV) and normal chest radiography. Patients with RVI symptoms, normal chest radiography, but a negative respiratory viral panel were enrolled as controls. Outcomes of interest included progression to LRTI within 30 days, oxygen requirement, and 30- and 90-day all-cause mortality.

**Figure 1:**
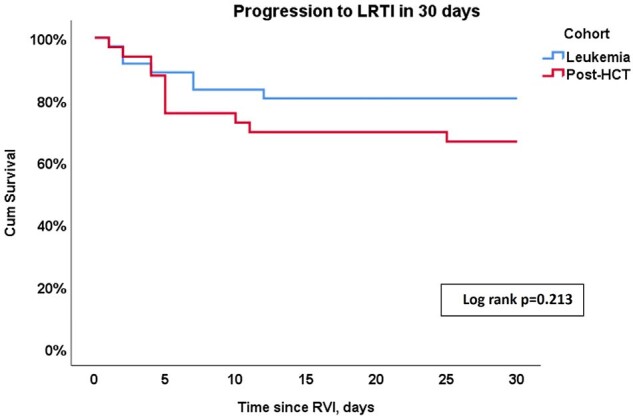
Progression to lower respiratory infection in 30 days of follow-up according to study cohort (leukemia vs. post-HCT) Abbreviations: LRTI, lower respiratory tract infection; HCT, hematopoietic cell transplant

**Results:**

Of 69 patients included, 36 (52%) had active leukemia and 33 (48%) were HCT recipients. Forty-eight (70%) patients had a diagnosis of RVI, including 17 (25%) RSV, 14 (20%) influenza, 17 (25%) PIV. We analyzed 21 (30%) control patients. During the follow-up period, 18 patients (11 HCT and 7 Leukemia) progressed to LRTI (Figure 1). In the LRTI group, we observed higher proportions of moderate and severe infections, based on maximal oxygen requirements within 30 days (p< 0.001), higher graft-versus-host disease diagnoses within 90 days following infection [5 (28%) vs 3 (6%), p=0.024, respectively], and increased 30- and 90-day all-cause mortality rates compared to patients to did not progress to LRTI [3 (17%) vs 0, p=0,016 and 5 (28%) vs 3 (6%), p=0.024, respectively]. Patients diagnosed with PIV were more likely to progress to LTRI within 30 days of diagnosis (Figure 2), but Influenza and RSV infections were associated with a higher 90-day all-cause mortality (Figure 3).

**Figure 2:**
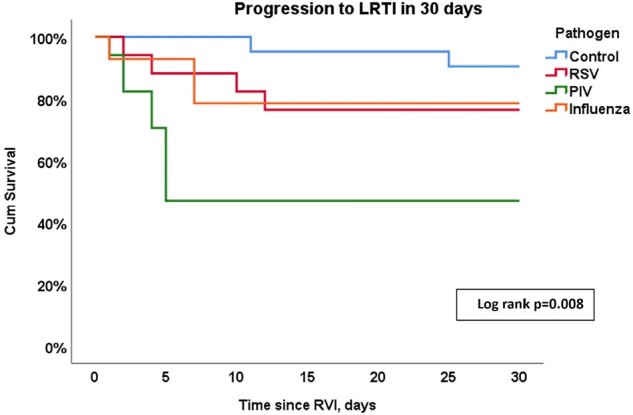
Progression to lower respiratory infection in 30 days of follow-up according to respiratory viral infection pathogen. Abbreviations: LRTI, lower respiratory tract infection; RSV, respiratory syncytial virus; PIV, parainfluenza

**Conclusion:**

HCT recipients and leukemia patients with PIV were more likely to progress to LRTI, but LRTI from influenza and RSV infections were associated with higher 30- and 90-day mortality.

**Figure 3:**
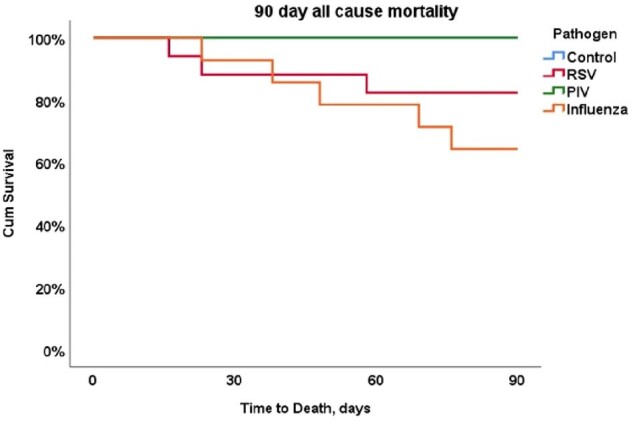
90-day mortality according to RVI pathogen. Abbreviation: RSV, respiratory syncytial virus; PIV, parainfluenza

**Disclosures:**

**Fareed Khawaja, MBBS**, Eurofins Viracor: Grant/Research Support|Symbio: Grant/Research Support **Kenneth J. Gollob, Ph.D.**, BMS: Honoraria|BMS: Gave a seminar on immuno-oncology|GSK: Grant/Research Support **Roy F. Chemaly, MD/MPH**, AiCuris: Advisor/Consultant|AiCuris: Grant/Research Support|Ansun Pharmaceuticals: Advisor/Consultant|Ansun Pharmaceuticals: Grant/Research Support|Astellas: Advisor/Consultant|Eurofins-Viracor: Grant/Research Support|InflaRX: Advisor/Consultant|Janssen: Advisor/Consultant|Karius: Advisor/Consultant|Karius: Grant/Research Support|Merck/MSD: Advisor/Consultant|Merck/MSD: Grant/Research Support|Moderna: Advisor/Consultant|Oxford Immunotec: Advisor/Consultant|Oxford Immunotec: Grant/Research Support|Roche/Genentech: Advisor/Consultant|Roche/Genentech: Grant/Research Support|Shinogi: Advisor/Consultant|Takeda: Advisor/Consultant|Takeda: Grant/Research Support|Tether: Advisor/Consultant

